# Efficacy and safety of systemic hydrocortisone for the prevention of bronchopulmonary dysplasia in preterm infants: a systematic review and meta-analysis

**DOI:** 10.1007/s00431-019-03398-5

**Published:** 2019-05-29

**Authors:** Ian Paul Morris, Nitin Goel, Mallinath Chakraborty

**Affiliations:** 1 Regional Neonatal Intensive Care Unit, University Hospital of Wales, Cardiff, CF14 4XW UK; 2 Centre for Medical Education, Cardiff University, Cardiff, UK

**Keywords:** Preterm, Infant, Bronchopulmonary dysplasia, Hydrocortisone, Steroid, Meta-analysis

## Abstract

Early lung inflammation has been implicated in the pathogenesis of bronchopulmonary dysplasia (BPD). We aimed to establish the efficacy and safety of systemic hydrocortisone for the prevention of BPD. A systematic review and meta-analysis were undertaken, with a detailed electronic literature search. Trials involving preterm infants were included if they were randomised to receive systemic hydrocortisone or a placebo. The primary outcome was the composite of survival without BPD at 36-week postmenstrual age (PMA). Results are presented as relative risk (RR) or risk difference (RD) with 95% confidence intervals (CIs), along with numbers needed to treat (NNT) or harm (NNH). After filtering, 12 studies using early (within 1 week of birth) and two using late hydrocortisone were identified. Early systemic hydrocortisone significantly increased the chances of survival without BPD (RR 1.13, 95% CI [1.01, 1.26], NNT 18), and survival without moderate-to-severe neurodevelopmental impairment (1.13 [1.02, 1.26], NNT 14). Infants who received hydrocortisone had a higher risk of intestinal perforation (1.69 [1.07, 2.68], NNH 30), primarily with concurrent treatment for patent ductus arteriosus.

*Conclusion*: Early systemic hydrocortisone is a modestly effective therapy for the prevention of BPD in preterm infants, although some safety concerns remain. No conclusions could be drawn for late hydrocortisone due to the paucity of studies.

**Table:** 

**What is Known:** • *Preterm infants are at high risk of developing bronchopulmonary dysplasia (BPD) and early lung inflammation plays a significant role in its pathogenesis.* • *Both early and late systemic dexamethasone seems to reduce the incidence of BPD, but its use is associated with serious neurodevelopmental impairment at follow-up.* **What is New:** • *Early systemic hydrocortisone significantly improved survival without BPD at 36 weeks and survival without moderate to severe neurodevelopmental impairment on follow up.* • *Incidence of gastrointestinal perforation associated with concurrent treatment for PDA was significantly higher, although early systemic hydrocortisone reduced the need for treatment of PDAs.*

**Electronic supplementary material:**

The online version of this article (10.1007/s00431-019-03398-5) contains supplementary material, which is available to authorized users.

## Introduction

Bronchopulmonary dysplasia (BPD) remains a common complication of preterm birth [1] and is associated with long-term pulmonary morbidity [2, 3] and neurodevelopmental impairment (NDI) [4]. Whilst BPD is multi-factorial in aetiology, persistent pulmonary inflammation beginning in utero and continued postnatally by factors including mechanical ventilation, oxidative stress and sepsis has been strongly implicated in the development of the disease [5]. Consequently, corticosteroids as potent anti-inflammatory agents could be of use in reducing the risk of developing BPD.

To date, most studies have considered systemic dexamethasone as the drug of choice in preventing or treating BPD [6, 7]. Benefits appear to include reduction in the need for mechanical ventilation, the incidence of BPD at 28 days and 36 weeks postmenstrual age (PMA), and neonatal mortality [6, 7]. However, concerns over long-term neurodevelopmental outcome, particularly when used within the first 7 days of life [6], have led to a more cautious approach in recent years [8] with dexamethasone use usually being reserved for those infants who are ventilator dependant beyond the first few weeks of life.

Systemic hydrocortisone has been postulated as a potentially safer drug to use in terms of long-term neurodevelopment [9, 10]. Several cohort studies have suggested no adverse effect on brain volume or neurodevelopmental outcome in infants receiving systemic hydrocortisone [9, 11], but prospective evidence supporting any benefit in facilitating extubation or reducing rates of BPD have been limited [12]. The Cochrane Neonatal Group recently updated a meta-analysis of efficacy and safety of systemic steroids in preterm infants, which included data on both hydrocortisone and dexamethasone. We conducted a specific and detailed systematic review and meta-analysis of systemic hydrocortisone to assess the efficacy of early (within the first week of life) or late (beyond the first week of life) postnatal use for the prevention of BPD in preterm infants compared to placebo or active control, along with its short- and long-term safety. Our analysis includes data from two extra studies, one using early hydrocortisone and a recent large study using late hydrocortisone. We have compared more clinically relevant outcomes (treated for hypertension, hyperglycaemia, patent ductus arteriosus [PDA] or retinopathy of prematurity [ROP], rather than incidence) to help clinicians in taking decisions on the ward. In addition, we have conducted a sub-group analysis of studies which had short-term respiratory endpoints as their primary outcome (BPD studies), excluding studies where hydrocortisone was used to treat hypotension, for more robust results.

## Methods

### Objectives

A systematic review and meta-analysis, using methods from the Cochrane Collaboration, to assess the efficacy and safety of systemic hydrocortisone for the prevention of BPD in preterm infants (< 37 weeks gestational age [GA] at birth), when compared with placebo (or other non-steroidal active control with no known effect on BPD) in published studies.

### Inclusion criteria

Prospective RCTs involving preterm infants were eligible for inclusion in the review. Trials were included if participating infants were randomised to receive systemic hydrocortisone (with or without a second active drug which has no known effects on BPD) started within the first week after birth (early) or after the first week (late), or a placebo (or any other non-steroidal active control with no known effects on BPD), and reported outcomes relevant to the review (please see below). Studies were grouped according to whether hydrocortisone was started early, or late, and separate analyses were conducted for each group.

### Search strategy

We developed a search strategy using keywords and MESH terms, as detailed in the supplementary information, from two main databases: Embase and Medline. A separate search using keyword was conducted on the Cochrane Central Register of Controlled Trials (CENTRAL). The databases were searched in March 2018, at the end of the third week. This search was rerun in February 2019, and one further relevant paper on late hydrocortisone was identified. References in included studies were also screened manually for inclusion. The search included papers in all languages from all countries.

### Outcomes

The primary outcome was survival without BPD at 36 weeks PMA (composite outcome). Data on several secondary outcomes were collected, including those on efficacy (survival to 36 weeks PMA and to discharge, and BPD at 36 weeks in survivors), short-term safety (sepsis, pulmonary air-leak or haemorrhage, gastrointestinal [GI] bleeding or perforation, hyperglycaemia and its treatment, hypertension and its treatment, intraventricular haemorrhage [IVH], periventricular leucomalacia [PVL] and necrotising enterocolitis [NEC]), other relevant short-term outcomes (home oxygen in survivors, duration of mechanical ventilation and total stay, patent ductus arteriosus [PDA] and its treatment, and retinopathy of prematurity [ROP] and its treatment) and long-term safety outcomes up to 2 years of age (death until last follow-up, survival without any NDI, survival without moderate-to-severe NDI, any and severe NDI at follow up and cerebral palsy [CP]).

### Definitions

BPD was defined as respiratory support and/or supplemental oxygen requirement at 36 weeks corrected GA and classified as moderate or severe BPD by Jobe and Bancalari. [13] Grades of IVH were as classified by Lu-Ann Papile [14]. Modified classification of NEC was by Walsh et al. [15]. PVL, PDA, ROP and NDI were defined as reported by authors in the studies.

### Data collection

Data was collected on characteristics of studies and planned outcomes using a standardised data collection form (supplementary Table 1) by at least two authors independently and then cross-checked for accuracy. Attempts were made to clarify methods and request additional data from corresponding authors if data on some relevant outcomes were not reported. These are mentioned in the relevant tables in the “Results” section.

### Statistical analysis

1. Measurement of Treatment Effect

Statistical analysis was conducted using Review Manager (RevMan) version 5.3 (Copenhagen: The Nordic Cochrane Centre, The Cochrane Collaboration, 2014). Only summary estimates are reported (no individual patient meta-analysis). For continuous outcomes, the mean and standard deviation (SD) (such as duration of respiratory support) reported in each study were collected and analysed and presented as mean differences (MD) along with 95% confidence intervals (CIs). Means and SDs were estimated from studies (total six studies) reporting continuous outcomes as medians and interquartile ranges by using methods described by Wan et al. [16]. For categorical outcomes (such as survival or BPD), data was extracted for each intervention group for analysis and presented as risk ratio (RRs) with 95% CI. Estimation of number needed to treat/harm, along with their 95% confidence intervals, were undertaken for significant results using GraphPad Prism QuickCalc (GraphPad software 2017, https://graphpad.com/quickcalcs/), according to the methods of Newcombe/Wilson with continuity correction [17]. All main results were rated independently by the authors using the Grade system (https://gdt.gradepro.org/app/handbook/handbook.html) and presented in a summary-of-findings (SoF) table. A pre-specified sub-group analysis was conducted by including studies in which hydrocortisone was used for the prevention of BPD (and not for the treatment of systemic hypotension). Significance was set at *p* < 0.05.

2. Assessment of Bias in Included Studies

All studies included in final analysis were assessed for risk of bias (low, high, or unknown) using a domain-based flow-sheet (as used by the Cochrane Collaboration). For each domain, a judgement was made on likely magnitude and direction of the bias and its likely impact on the outcomes. Disagreements were resolved by consensus. A judgement was made on the overall risk of bias based on the above domains.

3. Assessment of Heterogeneity

Heterogeneity was quantified using Inaccuracy^2^ (I^2^) statistic and stratified as moderate (I^2^ < 50%) or substantial (I^2^ ≥ 50%) (http://handbook.cochrane.org/). To calculate pooled estimate of effect size, a fixed-effect model was used if moderate heterogeneity was detected, and a random-effect model was used if substantial heterogeneity was detected.

### Ethical approval

No specific ethical approval was required for this meta-analysis as all original studies had individual ethical approval. The review was prospectively registered on PROSPERO with an identification number of CRD42017073615 (http://www.crd.york.ac.uk/PROSPERO/display_record.asp?ID=CRD42017073615).

## Results

**Fig. 1:**
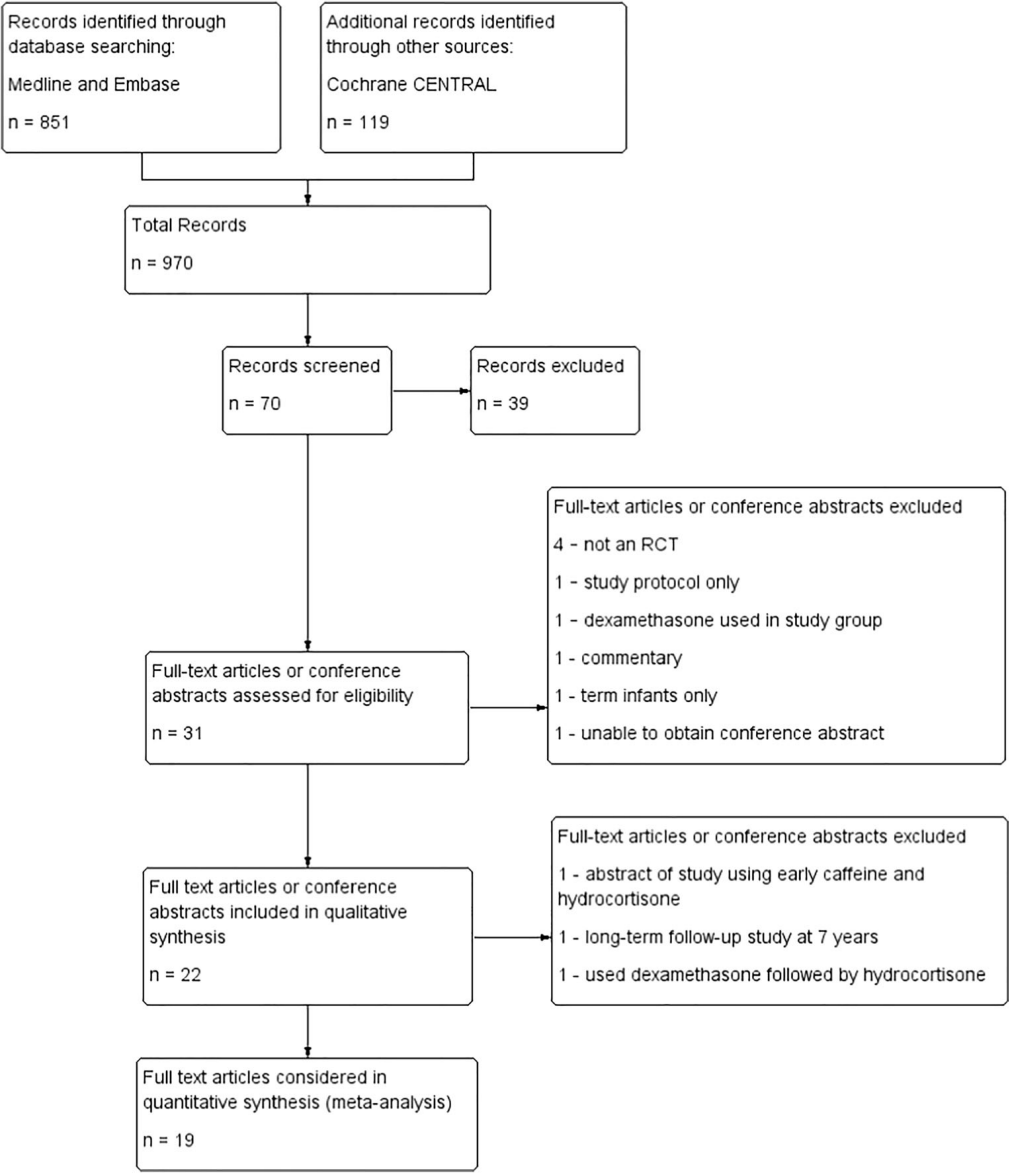
Study flow diagram, showing filtering of papers at each stage

Search records, filtering and study flow diagram is presented in Fig. 1. In total, 22 full-text articles and conference abstracts were included in the qualitative analysis—18 for the early use and 4 for the late use of hydrocortisone.

One full-text article and one conference paper for the early use and one full-text paper for the late use of hydrocortisone were excluded from final meta-analysis (details below), leaving 16 papers for the early use and 3 for the late use in the quantitative synthesis.

A summary of the risk of bias in the included studies as agreed by the authors is presented in supplementary Fig. 1. Although some of the domains in the included studies had unclear or high risk of bias, all the studies had an overall low risk of bias.

A total of 13 studies investigated the use of early hydrocortisone in preterm infants in the first week of life of which 12 were published as full-text articles [18–29]. One study was only published as an abstract [30] and used both hydrocortisone and caffeine in the experimental group, with placebo in the control group. As caffeine is known to have a significant effect on BPD, it was not possible to separate the effects of these two drugs from each other in the results from this study, and this abstract was excluded from further analysis. Of the 12 other studies which were included in the quantitative analysis, four published follow-up studies which were included in the long-term analysis [31–35]. One of the follow-up studies reported outcomes at pre-school age and was not included in the meta-analysis as this was the only study to do so [34]. Details of all included studies are presented in Table 1 and supplementary Table 2. Both the pooled mean GA (MD 0.05 weeks, 95% CI [− 0.09, 0.18], *p* = 0.49) and mean birth weight (− 3.92 g, [− 21.08, 13.24], *p* = 0.65) of the full cohort were comparable between the two groups of infants from the included studies.

**Table 1:** Characteristics of included studies

Author (year)	Participants	Study design	Timing of hydrocortisone: Early (≤7 days) or late (≥8 days)	Dates	Intervention participants	Control participants	Primary outcome
Baden (1972) [18]	Preterm infants	Randomised controlled trial	Early	Aug 1971–Apr 1972	*N* = 22	*N* = 22	Respiratory distress syndrome course and survival
Batton (2012) [19]	Preterm infants 23 + 0–26 + 6 weeks gestation	Randomised controlled trial	Early	Dec 2009–Dec 2010	*N* = 4	*N* = 6	Feasibility study (enrolment)
Baud (2016) [20]	Preterm infants 24 + 0–27 + 6 weeks gestation	Randomised controlled trial	Early	May 2008–Jan 2014	*N* = 255	*N* = 266	Survival without BPD at 36 weeks postmenstrual age
Baud (2017) [31]	2-year follow-up of Baud (2016)	Randomised controlled trial	Early	May 2008–Jan 2014	*N* = 255	*N* = 266	2-year neurodevelopmental outcome (secondary outcome)
Biswas (2003) [21]	Preterm infants < 30 weeks gestation	Randomised controlled trial	Early	Jan 1996–Apr 1998	*N* = 125	*N* = 128	Death or ventilator dependence at 1 week
Bonsante (2007) [22]	Preterm infants 24–30 weeks gestation	Randomised controlled trial	Early	Apr 2003–Sep 2005	*N* = 25	*N* = 25	Survival without oxygen at 36 weeks postmenstrual age
Bourchier (1997) [23]	Preterm infants < 1500 g birth weight	Randomised controlled trial	Early	Jul 1993–Jun 1995	*N* = 21	*N* = 25	Persistent hypotension despite treatment
Efird (2005) [24]	Preterm infants 23 + 0–28 + 6 weeks gestation	Randomised controlled trial	Early	May 2000–May 2002	*N* = 16	*N* = 18	Need for treatment for hypotension with vasopressin
Fitzhardinge (1974) [32]	1-year follow-up of Baden (1972) [18]	Randomised controlled trial	Early	1971–exact dates not specified	*N* = 13	*N* = 13	Respiratory distress syndrome course and survival
Hochwald (2014) [25]	Preterm infants ≤ 30 weeks gestation	Randomised controlled trial	Early	Jan 2007–Dec 2009	*N* = 11	*N* = 11	Vasopressor dose in hypotension
Ng (2006) [26]	Preterm infants < 32 weeks gestation	Randomised controlled trial	Early	Jun 2001–Nov 2004	*N* = 24	*N* = 24	Treatment of refractory hypotension
Onland 2019 [38]	Preterm infants < 30 weeks gestation and/or < 1250 g birth weight	Randomised controlled trial	Late	Nov 2011–Dec 2016	*N* = 181	*N* = 190	Death or BPD at 36 weeks postmenstrual age
Parikh (2013) [37]	Preterm infants ≤ 1000 g birth weight	Randomised controlled trial	Late	Oct 2005–Sep 2008	*N* = 31	*N* = 33	Total brain tissue volume as per MRI at 38 weeks postmenstrual age
Parikh (2015) [39]	18–22-month follow-up of Parikh (2013) [37]	Randomised controlled trial	Late	Oct 2005–Sep 2008	*N* = 31	*N* = 33	Neurodevelopmental outcomes and mortality at 18–22 months (secondary outcome)
Peltoniemi (2005) [27]	Preterm infants 23 + 0–30 + 0 weeks gestation	Randomised controlled trial	Early	Aug 2002–Mar 2004	*N* = 25	*N* = 26	Survival without oxygen at 36 weeks postmenstrual age
Peltoniemi (2009) [33]	2-year follow-up of Peltoniemi (2005) [27].	Randomised controlled trial,	Early	Aug 2002–Mar 2004	*N* = 25	*N* = 26	Growth and development at 2 years (secondary outcome)
Peltoniemi (2016) [34]	5–7-year follow-up of Peltoniemi (2005) [27].	Randomised controlled trial	Early	Aug 2002–Mar 2004	*N* = 25	*N* = 26	Growth and development at 5–7 years of age (secondary outcome)
Watterberg (1999) [28]	Preterm infants 500-999 g birth weight	Randomised controlled trial	Early	Jun 1996–May 1998	*N* = 20	*N* = 20	Survival without oxygen at 36 weeks postmenstrual age
Watterberg (2004) [29]	Preterm infants 500-999 g birth weight	Randomised controlled trial	Early	Nov 2001–Apr 2003	*N* = 180	*N* = 180	Survival without oxygen at 36 weeks postmenstrual age
Watterberg (2007) [35]	18–22-month follow-up of Watterberg (2004) [29]	Randomised controlled trial	Early	Nov 2001–Apr 2003	*N* = 180	*N* = 180	Growth and neurodevelopmental outcome at 18–22 months corrected age (secondary outcome)

**Fig. 2:**
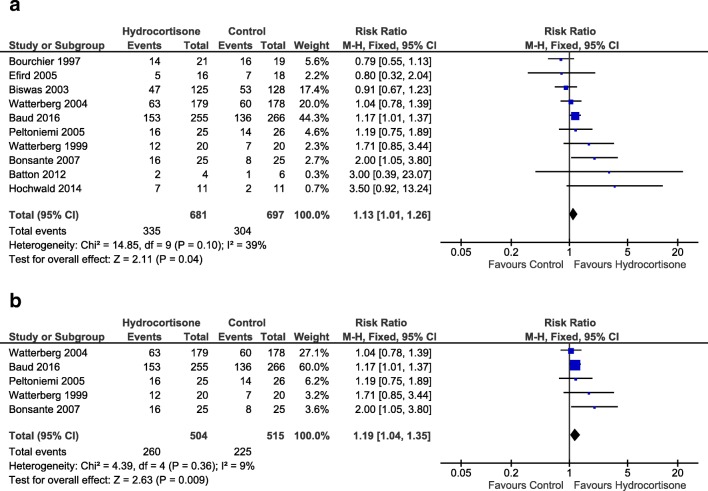
Forest-plot of pooled effect estimate for survival without BPD at 36 weeks corrected gestational age with early systemic hydrocortisone for **a** all studies and **b** studies with BPD as primary outcome

Summary of findings after early use of hydrocortisone is presented in Table 2. A total of 10 studies reported the primary outcome. Pooled estimate including data from all of the studies (1378 infants) showed a significantly higher risk of survival without BPD for the group of infants receiving hydrocortisone in the first week of life (RR 1.13 [1.01, 1.26], *p* = 0.04, Fig. 2a), compared to placebo (or other active control). From our estimate, 18 preterm infants would need to be treated (NNT) with early hydrocortisone for one infant to survive without BPD (95% CI 9.2, 314.2). A funnel plot for this outcome did not suggest a significant publication bias (supplementary Fig. 2a). As evident from Table 1, not all studies intended to look at the outcome of BPD; some studies used hydrocortisone to treat systemic hypotension in the first week of life [19, 21, 23–25]. We undertook a sub-group analysis of five studies (1019 infants) using early systemic hydrocortisone for the prevention of BPD as their primary outcome [20, 22, 27–29], and pooled data also showed a significantly higher risk of survival without BPD for infants in the hydrocortisone group (1.19 [1.04, 1.35], *p* < 0.01, Fig. 2b). Thirteen infants would have to be treated with early systemic hydrocortisone for one infant to survive without BPD (95% CI 7.1, 56.1).

**Table 2:** Summary of findings (SoF)

Early systemic hydrocortisone compared with control for preventing bronchopulmonary dysplasia in preterm infants						
Patient or population: chronic lung disease in preterm infants Setting: Neonatal Intensive Care Units Intervention: early systemic hydrocortisone Comparison: control						
Outcomes	Anticipated absolute effects^a^ (95% CI)		Relative effect (95% CI)	No. of participants (studies)	Certainty of the evidence (GRADE)	Comments
	Risk with control	Risk with early systemic hydrocortisone				
Survival without BPD at 36 weeks						
All studies	436 per 1000	493 per 1000 (441 to 550)	RR 1.13 (1.01 to 1.26)	1378 (10 studies)	MODERATE	
BPD studies	437 per 1000	520 per 1000 (454 to 590)	RR 1.19 (1.04 to 1.35)	1019 (5 studies)	HIGH	
BPD at 36 weeks in survivors						
All studies	460 per 1000	418 per 1000 (372 to 474)	RR 0.91 (0.81 to 1.03)	1198 (11 studies)	MODERATE	
BPD studies	458 per 1000	385 per 1000 (330 to 449)	RR 0.84 (0.72 to 0.98)	840 (5 studies)	HIGH	
Survival to 36 weeks						
All studies	818 per 1000	842 per 1000 (802 to 883)	RR 1.03 (0.98 to 1.08)	1347 (9 studies)	MODERATE	
BPD studies	807 per 1000	839 per 1000 (790 to 887)	RR 1.04 (0.98 to 1.10)	1022 (5 studies)	HIGH	
Gastrointestinal perforation						
All studies	47 per 1000	79 per 1000 (50 to 126)	RR 1.69 (1.07 to 2.68)	1099 (7 studies)	MODERATE	With concurrent treatment for PDA
BPD studies	47 per 1000	82 per 1000 (51 to 133)	RR 1.76 (1.09 to 2.84)	1017 (5 studies)	MODERATE	
Survival without moderate-severe NDI						
All studies	563 per 1000	636 per 1000 (574 to 709)	RR 1.13 (1.02 to 1.26)	898 (4 studies)	LOW	
BPD studies	565 per 1000	644 per 1000 (581 to 717)	RR 1.14 (1.03 to 1.27)	856 (3 studies)	LOW	

GRADE Working Group grades of evidence. High certainty: We are very confident that the true effect lies close to that of the estimate of the effect. Moderate certainty: We are moderately confident in the effect estimate: The true effect is likely to be close to the estimate of the effect, but there is a possibility that it is substantially different. Low certainty: Our confidence in the effect estimate is limited: The true effect may be substantially different from the estimate of the effect. Very low certainty: We have very little confidence in the effect estimate: The true effect is likely to be substantially different from the estimate of effect

^a^The risk in the intervention group (and its 95% confidence interval) is based on the assumed risk in the comparison group and the relative effect of the intervention (and its 95% CI), CI: Confidence Interval; RR: risk ratio

**Fig. 3:**
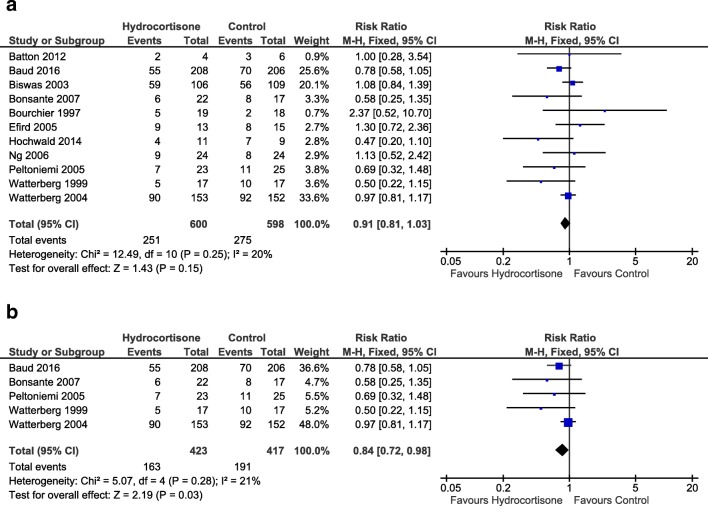
Forest plots of effect pooled estimates for BPD at 36 weeks corrected gestational age in survivors with early systemic hydrocortisone for **a** all studies and **b** studies with BPD as primary outcome

**Fig. 4:**
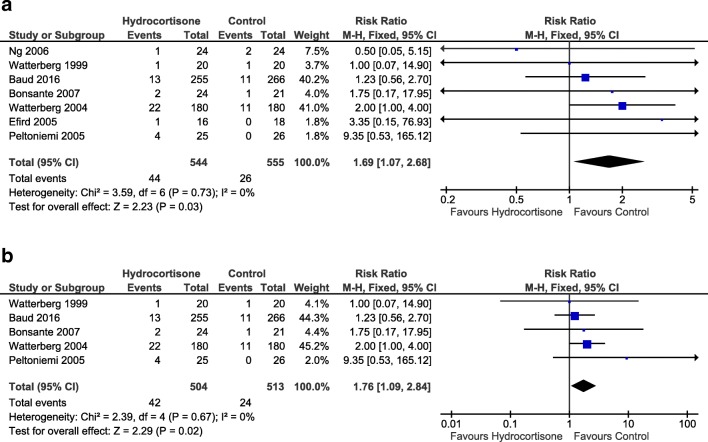
Forest plots of pooled effect estimates for gastrointestinal perforation with early systemic hydrocortisone for **a** all studies and **b** studies with BPD as primary outcome

When all studies were included, the incidence of BPD in survivors at 36 weeks (0.91 [0.81, 1.03], *p* = 0.15, Fig. 3a) and total survival to 36 weeks (1.03 [0.98, 1.08], *p* = 0.19) were not significantly different between the groups. However, in the sub-group analysis of the BPD studies, the risk of BPD in survivors at 36 weeks was significantly lower (0.84 [0.72, 0.98], *p* = 0.03, NNT 14 [7.2, 164.6], Fig. 3b), although survival to 36 weeks was comparable between the groups (1.04 [0.98, 1.10] *p* = 0.20). Total survival to discharge was higher in the hydrocortisone group (1.05 [1.00, 1.11], *p* = 0.04, NNT 24 [12.1, 524.2]) when all studies were included but failed to reach statistical significance in the sub-group analysis (1.06 [1.00, 1.13], *p* = 0.06). Gastrointestinal perforation, which was significantly higher in the group of infants receiving hydrocortisone (all studies: 1.69 [1.07, 2.68], *p* = 0.03, number needed to harm (NNH) 30 [15.9, 193.9], Fig. 4a; BPD-studies: 1.76 [1.09, 2.84], *p* = 0.02, NNH 28 [15.0, 159.2], Fig. 4b),

All short-term safety and other outcomes during the first admission of the infants from birth to discharge (or death) are summarised in Table 3 along with the sub-group analysis. Early treatment with hydrocortisone significantly reduced the risk of treatment for PDA (all-studies: 0.66 [0.52, 0.84], *p* < 0.01, NNT 11 [6.8, 25.9], supplementary Fig. 3a; BPD-studies: 0.66 [0.49, 0.88], *p* < 0.01, NNT 11 [6.3, 37.6], supplementary Fig. 3b).

**Table 3:** Summary of short-term safety and other outcomes during stay for use of early hydrocortisone

Outcome	Hydrocortisone		Control		Number of trials	Risk ratio (95% CI) OR SMD (95% CI)	*p* value	NNT/H (95% CI)
	Events	Total	Events	Total				
All studies								
Survival to discharge	602	728	583	743	12	1.05 (1.00, 1.11)	0.04	24 (12.1, 524.2)
Sepsis^a^	253	702	236	714	10	1.09 (0.95, 1.25)	0.22	
Pulmonary air leak	48	609	43	623	5	1.13 (0.77, 1.67)	0.54	
Pulmonary haemorrhage	53	584	41	598	4	1.32 (0.89, 1.95)	0.16	
GI bleeding	2	25	1	26	1	2.08 (0.20, 21.52)	0.54	
Treated for hyperglycaemia	207	508	192	520	6	1.10 (0.95, 1.28)	0.20	
Treated for hypertension	4	196	5	198	2	0.80 (0.22, 2.93)	0.74	
Any IVH	93	217	82	222	3	1.16 (0.92, 1.46)	0.20	
IVH grade III–IV^b^	102	687	108	704	10	0.97 (0.76, 1.25)	0.83	
PVL	21	584	30	607	8	0.75 (0.44, 1.27)	0.28	
NEC	54	705	55	719	11	1.00 (0.70, 1.43)	0.99	
Discharged with home oxygen (survivors)	33	348	43	341	4	0.76 (0.50, 1.15)	0.20	
Duration of mechanical ventilation (days)		425		430	8	−0.63 (−3.02, 1.77)	0.61	
Duration of stay (days)^c^		385		388	7	−1.94 (−6.76, 2.89)	0.43	
Treated for PDA^d^	79	439	125	458	5	0.66 (0.52, 0.84)	< 0.01	11 (6.8, 25.9)
Treated for ROP	25	477	30	486	5	0.83 (0.50, 1.37)	0.46	
BPD studies								
Survival to discharge	417	505	401	515	5	1.06 (1.00, 1.13)	0.06	
Sepsis	179	505	155	514	5	1.17 (0.99, 1.40)	0.07	
Pulmonary air leak	30	460	29	471	3	1.04 (0.64, 1.70)	0.86	
Pulmonary haemorrhage	37	435	30	446	2	1.26 (0.79, 2.01)	0.33	
GI bleeding	2	25	1	26	1	2.08 (0.20, 21.52)	0.54	
Treated for hyperglycaemia	196	460	184	472	3	1.09 (0.94, 1.28)	0.25	
Treated for hypertension	4	180	5	180	1	0.80 (0.22, 2.93)	0.74	
Any IVH	93	217	82	222	3	1.16 (0.92, 1.46)	0.20	
IVH grade III–IV	77	497	74	509	5	1.07 (0.80, 1.44)	0.65	
PVL	16	415	23	431	4	0.72 (0.39, 1.34)	0.30	
NEC	29	504	31	513	5	0.95 (0.58, 1.55)	0..84	
Discharged with home oxygen (survivors)	18	246	24	238	3	0.74 (0.42, 1.28)	0.28	
Duration of mechanical ventilation (days)		249		249	4	− 2.69 (− 6.98, 1.59)	0.22	
Duration of stay (days)		222		221	4	− 1.02 (− 6.35, 4.31)	0.71	
Treated for PDA^d^	54	300	85	312	3	0.66 (0.49, 0.88)	< 0.01	11 (6, 3, 37.6)
Treated for ROP	24	453	28	462	4	0.85 (0.51, 1.42)	0.54	

^a^Only first episode of sepsis included from Biswas 2003

^b^Bourchier (1997) and Efird (2005) reported IVH grades II–IV

^c^Efird (2005) reported duration of stay in survivors only

^d^Baud (2016) and Biswas (2003) reported numbers of infants who had surgery for PDA

Only a minority of the original studies reported any long-term outcomes (5 out of 11). The main results are summarised in Table 4. Infants who received early hydrocortisone had a significantly higher risk of survival without moderate-severe NDI (all studies: 1.13 [1.02, 1.26], *p* = 0.02, supplementary Fig. 4a; BPD-studies: 1.14 [1.03, 1.27], *p* = 0.02, supplementary Fig. 4b), compared to infants in the control group. For every 14 infants treated with early hydrocortisone after birth, one infant is estimated to survive without moderate-severe NDI (NNT, 95% CI 7.4, 129.4). If only the BPD studies were considered, the NNT was 13 (95% CI 7.0, 81.1).

**Table 4:** Summary of long-term safety outcomes of early hydrocortisone

Outcome	Hydrocortisone		Control		Number of trials	Risk ratio (95% CI)	*p* value	NNT (95% CI)
	Events	Total	Events	Total				
All studies								
Death until last FU	91	482	112	494	4	0.83 (0.65, 1.06)	0.14	
Survival without moderate-severe NDI	281	443	256	455	4	1.13 (1.02, 1.26)	0.02	14 (7.4, 129.4)
Any NDI at FU	41	138	45	138	2	0.91 (0.64, 1.29)	0.60	
Severe NDI at FU	19	217	26	207	2	0.70 (0.40, 1.22)	0.20	
Cerebral palsy (survivors and FU)	34	374	31	359	5	1.06 (0.67, 1.67)	0.81	
BPD studies								
Death until last FU	83	460	103	472	3	0.83 (0.64, 1.07)	0.15	
Survival without moderate-severe NDI	271	422	245	434	3	1.14 (1.03, 1.27)	0.02	13 (7.0, 81.1)
Any NDI at FU	39	126	44	126	1	0.89 (0.62, 1.26)	0.50	
Severe NDI at FU	19	217	26	207	2	0.70 (0.40, 1.22)	0.20	
Cerebral palsy (survivors and FU)	32	362	30	347	4	1.03 (0.64, 1.64)	0.91	

Results from Bonsante 2007 were reported in the paper by Peltoniemi 2009, Fitzhardinge reported severe NDI at 1-year of age, and Watterberg 2007 reported severe NDI)

*FU* follow-up

Three studies randomised infants to receive hydrocortisone after the first week of life [36–38], with one follow-up study [39]. The study by Kazzi et al. treated preterm infants with systemic dexamethasone in the first week of life (7 days), followed by systemic hydrocortisone in tapering doses for the next 10 days. As early dexamethasone is known to have a significant effect on BPD [6], it was not possible to separate its effect from those of hydrocortisone. Thus, this trial was excluded from further analysis. Summary of results from the remaining two trials are presented in Table 5. Although both of these studies used hydrocortisone in ventilator-dependent preterm infants beyond the first week of life for the prevention of BPD, there were several differences between their design and conduct. Parikh and colleagues [37] randomised infants with a birth weight ≤ 1000 g between 10 and 21 days of life to a cumulative dose of 17 mg/kg of hydrocortisone over 7 days or placebo, while Onland and colleagues [38] randomised infants born at < 30 weeks (or < 1250 g birth weight) between 7 and 14 days of life to a cumulative dose of 72.5 mg/kg of hydrocortisone over 22 days or placebo. Results reported by the two trials were not always comparable, and only some of the outcomes could be pooled together in the meta-analysis presented in Table 5. Apart from a significantly higher risk of needing treatment for hyperglycaemia in infants receiving hydrocortisone (2.31 [1.30, 4.11], *p* < 0.01), all other outcomes were comparable between the groups. There was a trend towards more infants in the hydrocortisone group surviving to discharge (1.12 [0.99, 1.26], *p* = 0.07). Due to the paucity of studies, no recommendations can be made for the use of late hydrocortisone for the prevention of BPD in preterm infants.

**Table 5:** Summary of outcomes of late hydrocortisone. (*FU* follow-up)

Outcome	Hydrocortisone		Control		Number of trials	Risk ratio (95% CI) SMD (95% CI)	*p* value
	Events	Total	Events	Total			
Survival without BPD at 36 weeks postmenstrual age	81	212	77	222	2	1.10 (0.88, 1.37)	0.41
BPD at 36 weeks in survivors	120	176	115	170	2	1.01 (0.88, 1.17)	0.87
Survival to 36 weeks postmenstrual age	176	212	170	223	2	1.09 (0.99, 1.20)	0.08
Survival to discharge	153	212	144	223	2	1.12 (0.99, 1.26)	0.07
Sepsis	104	212	126	223	2	0.87 (0.73, 1.04)	0.12
GI perforation	6	212	9	223	2	1.05 (0.11, 10.25)	0.96
Hypertension	27	212	26	223	2	1.10 (0.70, 1.73)	0.68
Treated for hyperglycaemia	33	181	15	190	1	2.31 (1.30, 4.11)	< 0.01
Treated for PDA	72	181	78	190	1	0.97 (0.76, 1.24)	0.80
IVH grade III–IV	2	181	3	190	1	0.70 (0.12, 4.14)	0.69
PVL	7	181	9	190	1	0.82 (0.31, 2.15)	0.68
NEC	19	212	22	223	2	0.91 (0.51, 1.63)	0.75
ROP > Grade II	44	181	42	190	1	1.10 (0.76, 1.59)	0.62
Duration of respiratory support		212		223	2	1.68 (−3.31, 6.67)	0.51
Death until last FU	9	29	12	29	1	0.75 (0.37, 1.50)	0.42
Survival without NDI (any)	9	28	7	29	1	1.33 (0.57, 3.09)	0.50
Cerebral palsy (survivors and FU)	3	20	1	17	1	2.55 [0.29, 22.31]	0.40

## Discussion

Preterm infants receiving early systemic hydrocortisone had a significantly higher chance of survival without BPD (composite outcome) at 36 weeks PMA, compared to control infants (NNT = 18). This effect was also evident in a sub-group of trials where BPD was the intended primary outcome with NNT of 13. Risk of gastrointestinal perforation was significantly higher in infants receiving hydrocortisone. At follow-up (up to 2 years of age), infants receiving early systemic hydrocortisone had a significantly higher chance of survival without moderate-to-severe NDI (composite outcome) compared to control infants (NNT 14).

Our review on hydrocortisone has been updated since the Cochrane review published in 2017, with several differences. We identified two extra eligible studies, which have been included in the meta-analysis. We have conducted a sub-group analysis with all studies where BPD was the primary outcome. In addition, we have looked at more clinically relevant outcomes which would help clinicians to take decisions on the ward, including proportions of infants in each group who actually received treatment for hyperglycaemia, hypertension, PDA and ROP. The diagnosis and the exact clinical significance of these morbidities are controversial; treatment for these morbidities indicate crossing of a pragmatic threshold, which is of real clinical interest (rather than just the diagnosis). Thus, we believe these clinically useful outcomes are more relevant for clinicians. We have used only published data for our analysis, which can be easily accessed and verified by all readers.

Our review includes all published trials of postnatal hydrocortisone (14 trials in total), including 1633 preterm infants. There is a modest but statistically significant increase in the chance of survival without BPD after receiving early postnatal hydrocortisone with an estimated NNT of 18 infants, although the imprecision of this estimate is acknowledged in the wide confidence interval. The sub-group analysis confirmed this effect with a smaller NNT of 13. Importantly, the follow-up studies, which included a total of 898 infants from 4 studies, demonstrated a significant increase in survival without moderate-to-severe NDI, with an NNT of 14. The incidence of cerebral palsy in survivors and NDI at follow-up was comparable between the two groups of infants. Though many of the studies did not report follow-up, the favourable neurodevelopmental results would be reassuring for clinicians that the long-term effects of early postnatal hydrocortisone use are in sharp contrast to those of early dexamethasone use in preterm infants [6]. However, we have only been able to analyse neurodevelopmental outcomes up to 2 years of age. One small study, involving 51 preterm infants, with longer-term follow up, have reported increasing trends towards NDI in infants receiving hydrocortisone at 5–7 years of age [34], suggesting collection of longer-term data would be prudent. Significantly more infants who received hydrocortisone survived to discharge (*p* = 0.04), although this outcome failed to reach statistical significance in the sub-group analysis (*p* = 0.06).

While the above outcomes are encouraging, use of early hydrocortisone resulted in a significantly higher risk of GI perforation, with an NNH of 30 infants. While hydrocortisone itself could cause intestinal perforation, an interaction between the steroid and indomethacin (or ibuprofen) has been strongly implicated as a significant contributor for this effect. Trials reporting increased incidence of GI perforation all used hydrocortisone with concurrent medical treatment for PDA [22, 26, 27, 29]. A similar interaction between early systemic dexamethasone and indomethacin were also noted in earlier studies [40]. Bourchier et al. [23] did not report an increased incidence of GI perforation, although this trial used the highest cumulative dose of early hydrocortisone but not in combination with PDA treatment. Use of ibuprofen for PDA was excluded from the PREMILOC trial in the first 24 h of life to avoid spontaneous GI perforations [20]. While these results generate serious concern, current clinical practice for managing PDAs are changing to a more conservative approach [41], since the spontaneous closure rate of PDAs remain high and early prophylactic treatment has failed to demonstrate significant clinical benefits. In addition, use of antenatal and postnatal steroids possibly decreases the incidence of PDA due to the inhibition of arachidonic acid and its metabolites, which have a significant effect on the patency of the duct [42].

The regime of early hydrocortisone was variable among the trials, but there were two broad groups: two trials used a total dose of ≥ 30 mg/kg but did not report any respiratory outcomes [18] or were primarily aimed for blood pressure management [23]; the rest used cumulative doses of ≤ 15 mg/kg including the five trials which used hydrocortisone for treatment of BPD. The chronic replacement dose of hydrocortisone in newborn infants, as recommended in the British National Formulary for Children (https://bnfc.nice.org.uk/drug/hydrocortisone.html), is 8–10 mg/m^2^/day, which is approximately equal to 1 mg/kg/day. The only appropriately powered trial, which had respiratory end-points as its primary outcome used this dose of hydrocortisone (cumulative dose 8.5 mg/kg over 10 days) and demonstrated significant clinical benefits [20]. While the most appropriate dose of hydrocortisone cannot conclusively be recommended from these results, the dose regime used by the PREMILOC trial seems to be safe and effective for survival without BPD in preterm infants. Four trials undertook formal adrenal stimulation tests after the course of hydrocortisone and reported no significant difference in cortisol levels, thus allaying fears of cortical suppression [20, 23, 27, 43].

Our review has several strengths. We have conducted a thorough electronic and manual search and believe that we have identified all published trials of hydrocortisone use in preterm infants, including two studies which are not included in the Cochrane review. Three authors have independently been involved in short listing and data collection, with joint cross-checking of all data and results. We have excluded trials where the individual effect of hydrocortisone could not be ascertained due to the use of concurrent drugs with known effects on BPD. Our analysis follows standard methods as recommended by the Cochrane collaboration (http://handbook-5-1.cochrane.org/). We have also looked at clinically relevant outcomes. Although many of the trials were not intended for the prevention of BPD but reported this outcome, we undertook a sub-group analysis by excluding these trials to reach robust conclusions. However, there are several limitations which are mostly related to the original studies. A number of trials in our analysis have included small numbers of infants increasing the chance of a type-I error [44], although they have all received lower weightage in the analyses. The increase in the incidence of gastrointestinal perforation with early systemic hydrocortisone remains of concern for clinicians, although this was associated mostly with concurrent treatment for PDA. However, this significant difference did not seem to have an overall effect in any other outcomes, including survival. In addition, early hydrocortisone seems to have a treatment-sparing effect on PDAs, which may mitigate some of this concern. While the long-term outcomes are reassuring, we acknowledge the reduction in confidence in this outcome due to incomplete follow-up in studies. The Premiloc study [20] used the Brunet-Lezine test to assess neurodevelopment at follow-up, while most of the other studies used variations of the Bailey’s test for this assessment. One study from Brazil, comparing these two assessment methods, reported moderate correlation between them in most domains but strong correlation in the language domain [45]. However, this limits our ability to interpret the results from the long-term outcomes.

## Conclusions

Early systemic hydrocortisone in preterm infants is effective in increasing the chances of survival without BPD at 36 weeks postmenstrual age. Incomplete long-term follow-up suggests significantly increased chance of survival without moderate-to-severe NDI up to 2 years of age, although the methods used to assess this outcome were inconsistent. An increased risk of GI perforation, mostly in conjunction with early treatment for PDA, remains of concern. Future trials should focus on ascertaining the most appropriate dose of early hydrocortisone in preterm infants for the prevention of BPD and undertake a longer period of follow-up to conclusively establish safety. In addition, recent trials of alternative delivery methods of early steroids have also shown encouraging results and should be further studied so that the optimum strategy to reduce BPD can be identified in these vulnerable infants.

Use of systemic hydrocortisone beyond the first week of life, especially in infants who become ventilator dependent, needs further research. Currently, one ongoing trial is recruiting infants in the second week of life to receive systemic hydrocortisone (https://clinicaltrials.gov/ct2/show/NCT01353313). Results from trials using late dexamethasone (https://www.npeu.ox.ac.uk/minidex) may also increase our knowledge in this area.

## Electronic supplementary material

ESM 1 (DOCX 327 kb)

ESM 2 (DOCX 23 kb)

## Abbreviations

BPD: Bronchopulmonary dysplasia
CENTRAL: Cochrane Central Register of Controlled Trials
CI: Confidence intervals
CP: Cerebral palsy
GA: Gestational age
GI: Gastrointestinal
IVH: Intraventricular haemorrhage
MD: Mean differences
MeSH: Medical Subject Headings
NDI: Neurodevelopmental impairment
NEC: Necrotising enterocolitis
NNH: Number needed to harm
NNT: Number needed to treat
PDA: Patent ductus arteriosus
PMA: Postmenstrual age
PVL: Periventricular leucomalacia
RCT: Randomised controlled trial
ROP: Retinopathy of prematurity
RR: Risk ratio
SD: Standard deviation
