# Supplementary material for: Efficacy and safety of systemic hydrocortisone for the prevention of bronchopulmonary dysplasia in preterm infants: a systematic review and meta-analysis
Source: Eur J Pediatr. 2019 May 29;178(8):1171–84. doi: 10.1007/s00431-019-03398-5 (PMC6647381; doi:10.1007/s00431-019-03398-5)
Supplement: Supplementary file 1 — (DOCX 327 kb) [file 431_2019_3398_MOESM1_ESM.docx]

**Efficacy and Safety of systemic hydrocortisone for the prevention of chronic lung disease: A Systematic Review and Meta-Analysis**

Ian P Morris, Nitin Goel, Mallinath Chakraborty

**Supplementary Information**

**E-text: Search Strategy and Details**

The following stepwise search strategy was used to look for relevant studies.

Database: EMBASE <1947-Present>, Ovid MEDLINE(R) ALL <1946 to February 01, 2019>

Search Strategy:

--------------------------------------------------------------------------------

1 exp Infant, Low Birth Weight/ (89416)

2 exp Infant, Very Low Birth Weight/ (23258)

3 exp Infant, Extremely Low Birth Weight/ (4636)

4 exp Infant, Premature/ (157619)

5 exp Premature Birth/ (117476)

6 Birth Weight/ (106750)

7 Low Birth Weight.mp. (92929)

8 ((Preterm* or Premature*) adj2 (labo#r* or birth* or born or infant or baby or babies or child or children or girl*1 or boy*1)).mp. (158831)

9 Prematurity.mp. (138473)

10 exp infant/ (2178593)

11 infant*.mp. (2149648)

12 newborn.mp. (1410486)

13 1 or 2 or 3 or 4 or 5 or 6 or 7 or 8 or 9 or 10 or 11 or 12 (2767651)

14 bronchopulmonary dysplasia/ (10467)

15 exp bronchopulmonary dysplasia/ (14762)

16 bronchopulmonary dysplasia.mp. (16185)

17 BPD/ (0)

18 exp BPD/ (0)

19 BPD.mp. (21078)

20 bronchopulmonary dysplasia.tw. (14422)

21 BPD.tw. (20892)

22 chronic lung disease/ (12103)

23 exp chronic lung disease/ (12103)

24 chronic lung disease.mp. (21746)

25 bronchopulmonary dysplasia.tw. (14422)

26 CLD/ (0)

27 CLD.mp. (7602)

28 CLD.tw. (7475)

29 14 or 15 or 16 or 17 or 18 or 19 or 20 or 21 or 22 or 23 or 24 or 25 or 26 or 27 or 28 (58253)

30 exp hydrocortisone/ (206565)

31 hydrocortisone.mp. (223390)

32 exp cortisone/ (57516)

33 cortisone.mp. (66698)

34 exp cortisol/ (206565)

35 cortisol.mp. (134928)

36 exp steroid/ (1559032)

37 steroid.mp. (494787)

38 exp corticosteroid/ (965174)

39 corticosteroid.mp. (341399)

40 30 or 31 or 32 or 33 or 34 or 35 or 36 or 37 or 38 or 39 (1931538)

41 Randomized Controlled Trials as Topic/ (214963)

42 randomized controlled trial/ (1012822)

43 Random Allocation/ (175061)

44 Double Blind Method/ (276262)

45 Single Blind Method/ (58167)

46 clinical trial/ (1489439)

47 controlled clinical trial.pt. (92892)

48 randomized controlled trial.pt. (475598)

49 multicenter study.pt. (244907)

50 clinical trial.pt. (514472)

51 exp Clinical Trials as topic/ (609573)

52 41 or 42 or 43 or 44 or 45 or 46 or 47 or 48 or 49 or 50 or 51 (2838714)

53 13 and 29 and 40 and 52 (986)

54 remove duplicates from 53 (851)

**Data Collection Form**

| **Source** | | **Y/N/not clear/not reported/comment** | | | | | |
| --- | --- | --- | --- | --- | --- | --- | --- |
| Study ID number | |  | | | | | |
| Report ID (surname of first author and year study undertaken) | |  | | | | | |
| Title | |  | | | | | |
| Authors names | |  | | | | | |
| Journal | |  | | | | | |
| Language published in | |  | | | | | |
| Reviewed by | |  | | | | | |
| Other comments | |  | | | | | |
| **Eligibility** | | | | | | | |
| Confirm eligibility for review | |  | | | | | |
| Gestation ≤ 37 weeks | |  | | | | | |
| Systemic hydrocortisone | |  | | | | | |
| **Reason for exclusion** | | | | | | | |
| Need to write to authors | |  | | | | | |
| Other comments | |  | | | | | |
| **Comparison** | | | | | | | |
| Hydrocortisone versus placebo | |  | | | | | |
| Hydrocortisone versus dexamethasone | |  | | | | | |
| Other (please state) | |  | | | | | |
| **Outcome data collected** | | | | | | | |
| Death (and time of death) | |  | | | | | |
| Death with other outcome (combined) - details | |  | | | | | |
| Total length of stay on neonatal unit (days) | |  | | | | | |
| Total days mechanical ventilation | |  | | | | | |
| Any air leaks | |  | | | | | |
| Pulmonary Haemorrhage | |  | | | | | |
| Respiratory support at 28 days | |  | | | | | |
| Respiratory support 36 weeks | |  | | | | | |
| Total duration of respiratory support | |  | | | | | |
| Home oxygen/respiratory support after discharge | |  | | | | | |
| IVH/PVL | |  | | | | | |
| Grade III/IV IVH/PVL | |  | | | | | |
| Neuro-developmental disability (any) – age | |  | | | | | |
| Infection/Sepsis | |  | | | | | |
| Significant Hypertension (requiring treatment) | |  | | | | | |
| Significant Hyperglycaemia (requiring treatment) | |  | | | | | |
| Necrotising enterocolitis | |  | | | | | |
| GI perforation | |  | | | | | |
| GI bleed | |  | | | | | |
| Retinopathy of prematurity (ROP) | |  | | | | | |
| Patent ductus arteriosus (PDA) | |  | | | | | |
| **Methods** | | | | | | | |
| Study design | |  | | | | | |
| Randomisation method | |  | | | | | |
| Blinding | |  | | | | | |
| Objective outcomes | |  | | | | | |
| Intention to treat analysis | |  | | | | | |
| **Participants** | | | | | | | |
| Total number | |  | | | | | |
| Total number in Hydrocortisone group | |  | | | | | |
| Total number in control group | |  | | | | | |
| Total number excluded/not included in analysis | |  | | | | | |
| Reason why excluded | |  | | | | | |
| CLD/BPD and how defined | |  | | | | | |
| **Assessment of Bias** | | | | | | | |
| Domain | | Risk of bias | | | | | |
|  | | **Low** | | **High** | **Unclear** | | |
| Sequence generation | |  | |  |  | | |
| Allocation concealment | |  | |  |  | | |
| Blinding | |  | |  |  | | |
| Detection | |  | |  |  | | |
| Attrition | |  | |  |  | | |
| Reporting | |  | |  |  | | |
| Other | |  | |  |  | | |
| **Overall risk of bias** | |  | |  |  | | |
| **Outcomes** | | | | | | |  |
| **Death during trial period** | | | **Hydrocortisone** | | **Control** | |  |
| Total number analysed in group | | |  | |  | |  |
| Events | | |  | |  | |  |
| **Death before discharge** | | |  | |  | |  |
| Total number analysed in group | | |  | |  | |  |
| Events | | |  | |  | |  |
| **Death by 28 days of age** | | |  | |  | |  |
| Total number analysed in group | | |  | |  | |  |
| Events | | |  | |  | |  |
| **Death by 36 weeks corrected gestation** | | |  | |  | |  |
| Total number analysed in group | | |  | |  | |  |
| Events | | |  | |  | |  |
| **Death/BPD at 28 days of age** | | | **Hydrocortisone** | | **Control** | |  |
| Total number analysed in group | | |  | | |  | |
| Events | | |  | | |  | |
| **Death/BPD at 36 weeks corrected gestational age** | | |  | | |  | |
| Total number analysed in group | | |  | | |  | |
| Events | | |  | | |  | |
| **Death/severe neurodisability** | | |  | | |  | |
| Total number analysed in group | | |  | | |  | |
| Events | | |  | | |  | |
| **Total days mechanical ventilation** | | |  | | |  | |
| Total number analysed in group | | |  | | |  | |
| Events | | |  | | |  | |
| **Respiratory support (any) at 28 days of age** | | |  | | |  | |
| Total number analysed in group | | |  | | |  | |
| Events | | |  | | |  | |
| **BPD at 36 weeks corrected gestational age** | | |  | | |  | |
| Total number analysed in group | | |  | | |  | |
| Events | | |  | | |  | |
| **Total duration of respiratory support (days)** | | |  | | |  | |
| Total number analysed in group | | |  | | |  | |
| Events | | |  | | |  | |
| **Total length of stay (days)** | | |  | | |  | |
| Total number analysed in group | | |  | | |  | |
| Events | | |  | | |  | |
| **Home oxygen therapy** | | |  | | |  | |
| Total number analysed in group | | |  | | |  | |
| Events | | |  | | |  | |
| **Any air leak (duration of trial)** | | | **Hydrocortisone** | | | **Control** | |
| Total number analysed in group | | |  | | |  | |
| Events | | |  | | |  | |
| **Any air leak (duration of stay)** | | |  | | |  | |
| Total number analysed in group | | |  | | |  | |
| Events | | |  | | |  | |
| **Pulmonary Haemorrhage** | | |  | | |  | |
| Total number analysed in group | | |  | | |  | |
| Events | | |  | | |  | |
| **IVH (any grade)** | | | **Hydrocortisone** | | | **Control** | |
| Total number analysed in group | | |  | | |  | |
| Events | | |  | | |  | |
| **IVH (grade III/IV)** | | |  | | |  | |
| Total number analysed in group | | |  | | |  | |
| Events | | |  | | |  | |
| **PVL** | | |  | | |  | |
| Total number analysed in group | | |  | | |  | |
| Events | | |  | | |  | |
| **PVL/IVH (any)** | | |  | | |  | |
| Total number analysed in group | | |  | | |  | |
| Events | | |  | | |  | |
| **PVL/IVH grade III/IV** | | |  | | |  | |
| Total number analysed in group | | |  | | |  | |
| Events | | |  | | |  | |
| **Severe neuro developmental delay (any def. at any time)** | | |  | | |  | |
| Total number analysed in group | | |  | | |  | |
| Events | | |  | | |  | |
| **Severe neuro developmental delay at 2 years (any def.)** | | |  | | |  | |
| Total number analysed in group | | |  | | |  | |
| Events | | |  | | |  | |
| **Gross motor delay (any def. at any time)** | | |  | | |  | |
| Total number analysed in group | | |  | | |  | |
| Events | | |  | | |  | |
| **Gross motor delay at 2 years (any def.)** | | |  | | |  | |
| Total number analysed in group | | |  | | |  | |
| Events | | |  | | |  | |
| **Necrotising enterocolitis** | | | **Hydrocortisone** | | | **Control** | |
| Total number analysed in group | | |  | | |  | |
| Events | | |  | | |  | |
| **GI perforation** | | |  | | |  | |
| Total number analysed in group | | |  | | |  | |
| Events | | |  | | |  | |
| **GI bleed** | | |  | | |  | |
| Total number analysed in group | | |  | | |  | |
| Events | | |  | | |  | |
| **Retinopathy of prematurity (any)** | | |  | | |  | |
| Total number analysed in group | | |  | | |  | |
| Events | | |  | | |  | |
| **Retinopathy of prematurity needing treatment** | | |  | | |  | |
| Total number analysed in group | | |  | | |  | |
| Events | | |  | | |  | |
| **Patent ductus arteriosus (any)** | | |  | | |  | |
| Total number analysed in group | | |  | | |  | |
| Events | | |  | | |  | |
| **Patent ductus arteriosus needing treatment** | | |  | | |  | |
| Total number analysed in group | | |  | | |  | |
| Events | | |  | | |  | |
| **Hypertension requiring treatment (duration of trial)** | | |  | | |  | |
| Total number analysed in group | | |  | | |  | |
| Events | | |  | | |  | |
| **Hypertension requiring treatment (duration of stay)** | | |  | | |  | |
| Total number analysed in group | | |  | | |  | |
| Events | | |  | | |  | |
| **Hyperglycaemia requiring treatment (duration of trial)** | | |  | | |  | |
| Total number analysed in group | | |  | | |  | |
| Events | | |  | | |  | |
| **Hyperglycaemia requiring treatment (duration of stay)** | | |  | | |  | |
| Total number analysed in group | | |  | | |  | |
| Events | | |  | | |  | |
| **Sepsis** | | |  | | |  | |
| Total number analysed in group | | |  | | |  | |
| Events | | |  | | |  | |
| **Miscellaneous** | | | | | | | |
| Funding source |  | | | | | | |
| Key conclusions |  | | | | | | |
| Miscellaneous comments from authors |  | | | | | | |
| References to other relevant studies |  | | | | | | |
| Other comments |  | | | | | | |

Supplementary Table 1: Data collection form

| **Author (Year)** | **Study design** | **Timing of hydrocortisone: Early (≤7 days) or late (≥ 8 days)** | **Participants** | **Intervention and control groups** | **Outcome measures** |
| --- | --- | --- | --- | --- | --- |
| Baden (1972) | Randomised controlled trial, blinded, of hydrocortisone versus lactose placebo in the management of RDS | Early | All infants diagnosed with RDS based on clinical, blood gas, and radiological features.  Exclusions: None stated | Infants randomly assigned to one of two groups:   1. Hydrocortisone 15mg/kg 2. Equivalent volume of lactose / diluents   Hydrocortisone dose 15mg/kg at admission and at 12 hours  Total dose 30mg/kg | Primary outcome unclear.  Studied outcomes:  Plasma corticosteroid concentrations  72-hour blood gas and oxygen parameters  Death before discharge  NEC |
| Batton (2012) | Randomised controlled trial, blinded, comparing the effects of hydrocortisone, dopamine and placebo on blood pressure | Early | 23+0 – 26+6 weeks gestation with protocol-defined low blood pressures. 7 US centres.  Exclusions: Infants with a major birth defect, a decision to withhold or withdraw care, absence of umbilical arterial catheter, or received > 20ml/kg fluid boluses, indomethacin, or ibuprofen before enrollment. | Infants randomly assigned to receive a study infusion and a study syringe medication, thus allocated to one of four groups:   1. Dopamine / placebo 2. Dopamine / hydrocortisone 3. Placebo / placebo 4. Placebo / hydrocortisone   Hydrocortisone dose of an initial 1mg/kg followed by 6 doses of 0.5mg/kg every 12 hours  Total dose: 4mg/kg | Pilot study to assess feasibility.  Secondary outcomes:  BPD (not defined)  Grade III/IV IVH  PVL  NEC  Survival to hospital discharge |
| Baud (2016) | Randomised controlled trial, blinded, comparing hydrocortisone versus placebo in improving survival without BPD | Early | Inborn 24+0 – 27+6 weeks gestation in 21 French centres, day 1 of life.  Exclusions: rupture of membranes before 22 weeks, birth-weight < 3^rd^ centile, severe perinatal asphyxia, expected to die shortly after birth, congenital malformations or known chromosomal aberrations. | Infants randomly assigned to one of two groups:   1. Hydrocortisone 2. 5% glucose   Hydrocortisone dose 0.5mg/kg BD for 7 days, 0.5mg/kg OD for 3 days  Total dose 8.5mg/kg | Primary outcome:  Survival without BPD (oxygen or ventilator support at 36 weeks, with physiological definition) at 36 weeks post-menstrual age  Secondary outcomes: BPD, death, surgical ligation of PDA. Adverse events (air leaks, pulmonary haemorrhage, insulin requirement, NEC, GI perforation, severe brain damage, severe ROP) |
| Baud (2017) | Randomised controlled trial, blinded, comparing hydrocortisone versus placebo in improving survival without BPD | Early | 2-year follow-up of Baud (2016) to assess neurodevelopmental outcomes | Infants randomly assigned to one of two groups:   1. Hydrocortisone 2. 5% glucose   Hydrocortisone dose 0.5mg/kg BD for 7 days, 0.5mg/kg OD for 3 days  Total dose 8.5mg/kg | Secondary outcome:  Neurodevelopmental impairment based on standardized neurological examination and revised Brunet-Lezine scale |
| Biswas (2003) | Randomised controlled trial, blinded, comparing hydrocortisone plus tri-iodothyronine (T_3_) versus placebo in decreasing mortality and respiratory morbidity | Early | <30 weeks gestation, mechanically ventilated within first 5 hours. 4 UK centres.  Exclusions: any major morphological abnormality | Infants randomly assigned to one of two groups:   1. Hydrocortisone plus T3 2. 5% dextrose   Hydrocortisone dose 1mg/kg as a continuous infusion within 5 hours, for 5 days and then 0.5mg/kg/day for 2 days  Total dose 6mg/kg | Primary outcome:  Death or ventilator dependence at 7 days, death or oxygen dependence at 2 weeks.  Secondary outcomes:  Duration of ventilation  Duration of oxygen support  Oxygen dependence at 36 weeks Duration of hospital stay  IVH  PDA  Sepsis |
| Bonsante (2007) | Randomised controlled trial, blinded, comparing hydrocortisone versus placebo in improving survival without BPD | Early | 24-30 weeks, 500-1249g at birth, requiring mechanical ventilation after surfactant administration, < 48 hours old. 2 centres Italy.  Exclusions: cardiopulmonary malformations, perinatal asphyxia, death within 12 hours of recruitment, use of steroids before and during treatment period | Infants randomly assigned to one of two groups:   1. Hydrocortisone 2. Normal saline   Hydrocortisone dose 0.5mg/kg BD for 9 days, then 0.5mg/kg OD for 3 days  Total dose 10.5mg/kg | Primary outcome:  Survival without oxygen at 36 weeks  Secondary outcomes:  Hypotension  Death before discharge  Duration of mechanical ventilation  PDA requiring treatment  ROP (stage 3 or 4 needing treatment)  IVH grade III or IV  PVL  Dexamethasone for severe CLD  Adverse effects (NEC, hypertension, GI perforation, insulin requirement, sepsis, cerebral palsy at 2 years) |
| Bourchier (1997) | Randomised controlled trial, comparing hydrocortisone with dopamine for the treatment of hypotension | Early | <1500g, < 7 days old, requiring inotropic support for hypotension. Single centre, New Zealand.  Exclusions: major congenital anomaly, shock, clinically significant PDA (LA:Ao >1.5 or diameter > 1.5mm) after 3 days. | Infants randomly assigned to one of two groups:   1. Dopamine 2. Hydrocortisone   Hydrocortisone dose 2.5mg/kg at 0 and 4 hours, then 6 hourly for 48 hours. Then 1.25mg/kg QDS for 48 hours, then 0.625mg/kg QDS for 48 hours.  Total dose 37.5mg | Primary outcome:  Persistent hypotension  Secondary outcome:  Correlation between plasma cortisol concentrations and response to treatment  Survival  IVH  BPD (oxygen at 36 weeks)  ROP  Sepsis |
| Efird (2005) | Randomised controlled trial, blinded, to compare hydrocortisone versus placebo in preventing hypotension | Early | 23+0 – 28+6 weeks gestation, inborn, 501-999g, within 2 hours of birth. Single US centre.  Exclusions: major malformations, chromosomal abnormalities, congenital heart disease. | Infants randomly assigned to one of two groups:   1. Normal saline 2. Hydrocortisone   Hydrocortisone dose 1mg/kg BD for 2 days, then 0.3mg/kg BD for 3 days.  Total dose 5.8mg/kg | Primary outcome:  Hypotension requiring treatment  Secondary outcome:  Days requiring vasopressors  Serum cortisol levels  BPD (oxygen at 36 weeks)  Sepsis  NEC  GI perforation  PDA  IVH  PVL  Duration of hospital stay |
| Fitzhardinge (1974) | Randomised controlled trial, blinded, of hydrocortisone versus placebo in the management of RDS | Early | 1-year follow-up of Baden (1972) | Infants randomly assigned to one of two groups:   1. Hydrocortisone 15mg/kg 2. Equivalent volume of lactose / diluents   Hydrocortisone dose 15mg/kg at admission and at 12 hours  Total dose 30mg/kg | Secondary outcome:  Growth, development and immune competence until 1 year of age |
| Hochwald (2014) | Randomised controlled trial, blinded, to compare hydrocortisone versus placebo in treating hypotension | Early | ≤30 weeks gestation, ≤1250g, within 48 hours of birth and hypotensive refractory to normal saline boluses. Single centre, Israel.  Exclusions: blood loss, hydrops, major cardiac lesions | Infants randomly assigned to one of two groups:   1. Normal saline + dopamine 2. Hydrocortisone + dopamine   Hydrocortisone dose 2mg/kg, then 3 x 1mg/kg 6 hourly, then 4 x 0.5mg/kg 6 hourly.  Total dose 7mg/kg | Primary outcome:  Reduction in inotrope use  Secondary outcome:  BPD (oxygen at 36 weeks)  Death  Sepsis  NEC  Duration of respiratory support  Duration of oxygen  Insulin requirement |
| Kazzi (1990) | Randomised controlled trial, blinded, to compare dexamethasone then hydrocortisone versus placebo in managing BPD | Late | <1500g, radiological evidence of BPD, ventilator dependent and in ?35% oxygen at 4 weeks. Single US centre.  Exclusions: clinically significant PDA, pneumonia, sepsis, hypertension. | Infants randomly assigned to one of two groups:   1. Normal saline 2. Dexamethasone and hydrocortisone   Dexamethasone 0.5mg/kg/day for 3 days, 0.4mg/kg/day for 3 days, then 0.25mg/kg/day for 2 days.  Hydrocortisone then given at 2mg/kg QDS for 2 days, reducing by 50% alternate days until 0.5mg/kg/day (10 days total)  Total dose 31mg/kg | Primary outcome:  Extubation within 8 days of dexamethasone  Secondary outcome:  Days on oxygen  Days mechanical ventilation  Duration of hospital stay  Home oxygen  ROP  Growth |
| Ng (2007) | Randomised controlled trial, blinded, to compare hydrocortisone versus placebo in preventing hypotension | Early | < 32 weeks gestation, <1500g, hypotension requiring dopamine in first 7 days of life. Single centre, Hong Kong  Exclusions: major or lethal congenital anomalies, congenital heart defects (excluding PDA), postnatal corticosteroids for BPD, sepsis, NEC or major surgery before randomization. | Infants randomly assigned to one of two groups:   1. Isotonic saline 2. Hydrocortisone   Hydrocortisone dose 1mg/kg TDS for days  Total dose 15mg/kg | Primary outcome:  Ability to wean off vasopressor support within 72 hours of treatment  Secondary outcome:  Duration of respiratory support  BPD (oxygen at 36 weeks)  Death  Duration  Adverse effects (NEC, sepsis, GI perforation, pulmonary haemorrhage, IVH, PVL, air leak, ROP) |
| Onland (2013) | Randomised controlled trial, blinded, to assess the effect of hydrocortisone initiated between 7 and 14 days after birth on death or BPD in very preterm infants. | Late | Infants born at a gestational  age of less than 30 weeks and/or with a birth weight of less  than 1250 g who were ventilator dependent between 7 and  14 days’ postnatal age and at high risk of developing BPD  were eligible. High risk of developing BPD was defined as having a respiratory index (product of mean airway pressure and the fraction of inspired oxygen) equal to or greater  than 3.5 for more than 12 h/d for at least 48 hours.  Infants  were ineligible if they had chromosomal defects or major  congenital malformations or had received corticosteroids  for improving lung function in the first week of life. | Eligible infants were randomly allocated in a 1:1 ratio to either  hydrocortisone (hydrocortisone sodium succinate, 5mg/kg per day in 4 doses per day for 7 days, followed by 3.75 mg/kg per day in 3 doses per day for 5 days, subsequently lowering the frequency by 1 dose every 5 days, resulting in 22 days of treatment with a cumulative dose of 72.5mg/kg) or placebo (mannitol), stratified by study center and gestational  age (<27 weeks or =27 weeks) and using randomly permuted block sizes between 2 and 8. | Primary outcome:  Composite of death or BPD at 36  weeks’ postmenstrual age.  Secondary outcome:  Mortality at hospital discharge, failure to extubate at days 3, 7, 14, and 21, total duration of mechanical ventilation and supplemental oxygen,  hospital length of stay,  necrotizing enterocolitis, gastrointestinal bleeding, spontaneous  intestinal perforation, intraventricular hemorrhage,  periventricular leukomalacia, retinopathy of prematurity,  hypertension, hyperglycemia, sepsis, patent ductus arteriosus, and growth at 36 weeks’ postmenstrual age. |
| Parikh (2013) | Randomised controlled trial, blinded, to compare hydrocortisone versus placebo effects on brain volumes | Late | ≤1000g, ventilator dependent between 10 and 21 days. Single US centre.  Exclusions: <23 weeks gestation, previous postnatal corticosteroids, received indomethacin, sepsis, NEC, major congenital anomaly of cardiopulmonary or CNS. | Infants randomly assigned to one of two groups:   1. Normal saline 2. Hydrocortisone   Hydrocortisone dose 1.5mg/kg BD for 4 days, then 1mg/kg BD for 2 days, then 0.5mg/kg BD for 1 day  Total dose 17mg/kg | Primary outcome:  Total brain tissue volume at 38 weeks postmenstrual age  Secondary outcome:  Survival without severe BPD  Duration positive pressure support  Days on oxygen  Adverse events (hypertension, sepsis, GI perforation or bleed) |
| Parikh (2015) | Randomised controlled trial, blinded, to compare hydrocortisone versus placebo effect on survival without neurodevelopmental impairment | Late | 18-22-month follow-up of Parikh (2013). Single centre US. | Infants randomly assigned to one of two groups:   1. Normal saline 2. Hydrocortisone   Hydrocortisone dose 1.5mg/kg BD for 4 days, then 1mg/kg BD for 2 days, then 0.5mg/kg BD for 1 day  Total dose 17mg/kg | Primary outcome:  Death  Survival without neurodevelopmental impairment (cerebral palsy, severe hearing or visual loss, cognitive or language delay)  Secondary outcomes:  Need for respiratory therapies  Somatic growth |
| Peltoniemi (2005) | Randomised controlled trial, blinded, to compare hydrocortisone versus placebo effect on survival without BPD | Early | 23+0 – 30+0, 501-1250G, requiring oxygen and mechanical ventilation beyond 24 hours. 3 centres, Finland.  Exclusions: lethal malformations or suspected chromosomal abnormalities. | Infants randomly assigned to one of two groups:   1. Isotonic saline 2. Hydrocortisone   Hydrocortisone dose 2.0mg/kg over 3 doses 8 hourly for 2 days, then 1.5mg/kg over 3 doses 8 hourly for 2 days, then 0.75mg/kg over 2 doses 12 hourly for 6 days  Total dose 11.5mg/kg | Primary outcome:  Survival without BPD (oxygen at 36 weeks)  Secondary outcome:  IVH grades III-IV  Cystic PVL  Ventilatory and oxygen support at 14 and 28 days and 36 weeks  Duration mechanical ventilation  Duration hospital stay  Sepsis  NEC  PDA needing treatment  ROP  Adverse events (insulin requirement, hypo or hypertension, GI bleed or perforation) |
| Peltoniemi (2009) | Randomised controlled trial, blinded, to compare hydrocortisone versus placebo effect on neurodevelopmental outcome | Early | 2-year follow-up of Peltoniemi (2005). | Infants randomly assigned to one of two groups:   1. Isotonic saline 2. Hydrocortisone   Hydrocortisone dose 2.0mg/kg over 3 doses 8 hourly for 2 days, then 1.5mg/kg over 3 doses 8 hourly for 2 days, then 0.75mg/kg over 2 doses 12 hourly for 6 days  Total dose 11.5mg/kg | Secondary outcome:  Growth  Neurodevelopmental impairment at 2 years - cerebral palsy, cognitive development, major neurosensory impairment, abnormal speech |
| Peltoniemi (2016) | Randomised controlled trial, blinded, to compare hydrocortisone versus placebo effect on neurodevelopmental outcome | Early | 5-7-year follow-up of Peltoniemi (2005). | Infants randomly assigned to one of two groups:   1. Isotonic saline 2. Hydrocortisone   Hydrocortisone dose 2.0mg/kg over 3 doses 8 hourly for 2 days, then 1.5mg/kg over 3 doses 8 hourly for 2 days, then 0.75mg/kg over 2 doses 12 hourly for 6 days  Total dose 11.5mg/kg | Secondary outcome:  Neurodevelopmental outcome at preschool age |
| Watterberg (1999) | Randomised controlled trial, blinded, to compare hydrocortisone versus placebo effect on survival without BPD | Early | 500-999g, within 48 hours of birth, mechanically ventilated. 2 US centres. Pilot study.  Exclusions: maternal diabetes, congenital sepsis, small for gestational age. | Infants randomly assigned to one of two groups:   1. Normal saline 2. Hydrocortisone   Hydrocortisone dose 0.5mg/kg BD for 9 days, then 0.5mg/kg OD for 3 days  Total dose 10.5mg/kg | Primary outcome:  Survival without BPD (oxygen at 36 weeks)  Secondary outcome:  Duration mechanical ventilation  Duration oxygen >40% or >25%  Home oxygen  Duration of stay  Growth at 36 weeks  Sepsis  NEC  PDA  IVH  ROP |
| Watterberg (2004) | Randomised controlled trial, blinded, to compare hydrocortisone versus placebo effect on survival without BPD | Early | 500-999g, mechanically ventilated between 12 and 48 hours of life.  Exclusions: major congenital anomaly, congenital sepsis, postnatal glucocorticoid other than hydrocortisone, triplet or higher order pregnancy | Infants randomly assigned to one of two groups:   1. Normal saline 2. Hydrocortisone   Hydrocortisone dose 0.5mg/kg BD for 12 days, then 0.5mg/kg OD for 3 days  Total dose 13.5mg | Primary outcome:  Survival without BPD (oxygen at 36 weeks)  Secondary outcome:  Death before 36 weeks  Death before discharge  BPD in survivors  Duration mechanical ventilation  Oxygen therapy  Duration of stay  Growth at 36 weeks  Sepsis  NEC  PDA  IVH  ROP |
| Watterberg (2007) | Randomised controlled trial, blinded, to compare hydrocortisone versus placebo effect on growth and neurodevelopmental outcome | Early | 18-22-month follow-up of Watterberg (2004) | Infants randomly assigned to one of two groups:   1. Normal saline 2. Hydrocortisone   Hydrocortisone dose 0.5mg/kg BD for 12 days, then 0.5mg/kg OD for 3 days  Total dose 13.5mg | Secondary outcome:  Neurodevelopmental impairment at 18-22 months corrected age |

Supplementary Table 2: Clinical details of included studies


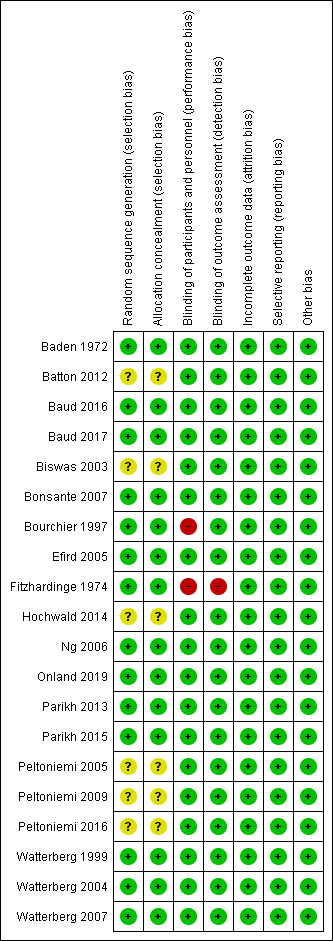


Supplementary Figure 1: Risk of bias summary: review authors' judgements about each risk of bias item for each included study.

A


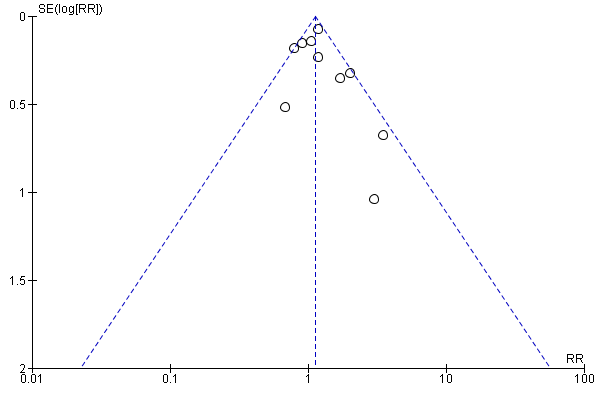


B


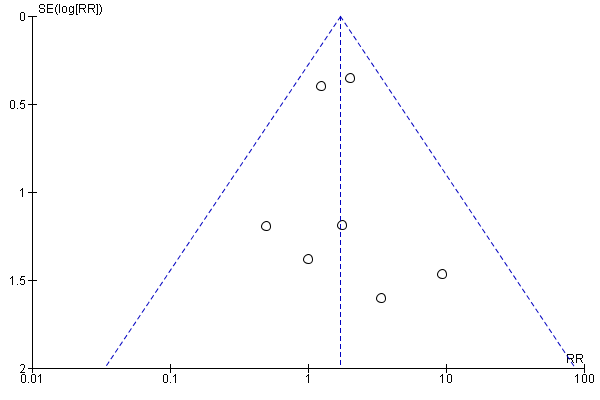


C


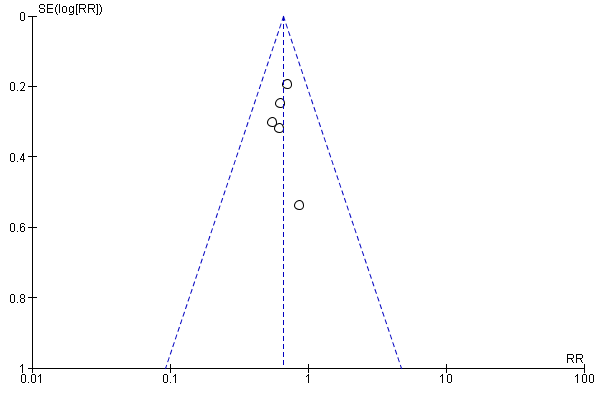


D


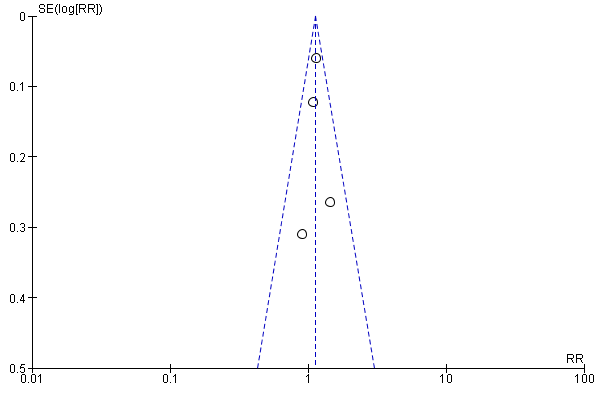


Supplementary Figure 2: Funnel plots of effect estimate (RR on x-axis) of (a) survival without BPD (b) GI perforation, (c) treatment for PDA, and (d) survival without moderate-severe NDI, against their respective standard errors (on y-axis). Points plotted are individual studies.

A


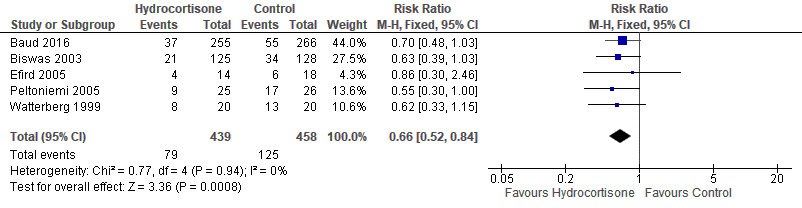


B


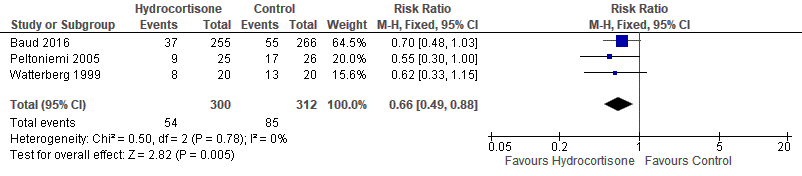


Supplementary Figure 3: Forest-plot of pooled effect estimate of treatment for PDA (A) all studies, and (B) studies with BPD as primary outcome.

A


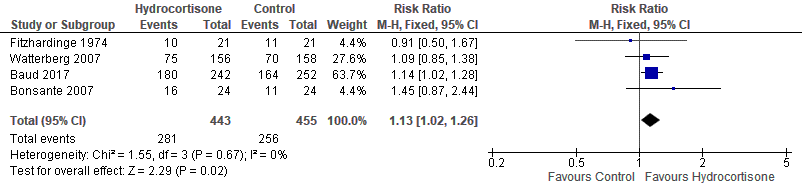


B


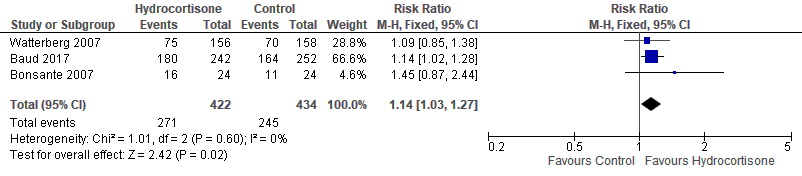


Supplementary Figure 4: Forest-plot of pooled effect estimate of survival without moderate to severe NDI (A) all studies, and (B) studies with BPD as primary outcome.
